# Supplementary material for: Bereavement care for ethnic minority communities: A systematic review of access to, models of, outcomes from, and satisfaction with, service provision
Source: PLoS One. 2021 Jun 30;16(6):e0252188. doi: 10.1371/journal.pone.0252188 (PMC8244918; doi:10.1371/journal.pone.0252188)
Supplement: S2 File — (DOCX) [file pone.0252188.s003.docx]

**S2 File: MMAT quality appraisal summary**

| **Study** | **MMAT tool** | **Responses** | | |
| --- | --- | --- | --- | --- |
| Ackroyd, R. (2003). "Audit of referrals to a hospital palliative care team: role of the bilingual health-care worker." International journal of palliative nursing 9(8): 352-357. | ***Quantitative descriptive*** | yes | no | can't tell |
|  | 4.1. Is the sampling strategy relevant to address the research question? | Yes |  |  |
|  | 4.2. Is the sample representative of the target population? | Yes |  |  |
|  | 4.3. Are the measurements appropriate? | Yes |  |  |
|  | 4.4. Is the risk of nonresponse bias low? | N/A |  |  |
|  | 4.5. Is the statistical analysis appropriate to answer the research question? | Yes |  |  |
| Koffman, J., N. Donaldson, M. Hotopf and I. J. Higginson (2005). "Does ethnicity matter? Bereavement outcomes in two ethnic groups living in the United Kingdom." Palliative & supportive care 3(3): 183-190. | ***Quantitative non-randomized studies*** | yes | no | can't tell |
|  | 3.1. Are the participants representative of the target population? | Yes |  |  |
|  | 3.2. Are measurements appropriate regarding both the outcome and intervention (or exposure)? | Yes |  |  |
|  | 3.3. Are there complete outcome data? | Yes |  |  |
|  | 3.4. Are the confounders accounted for in the design and analysis? | Yes |  |  |
|  | 3.5 During the study period, is the intervention administered (or exposure occurred) as intended? | Yes |  |  |
| Koffman, J. and I. J. Higginson (2002). "Religious faith and support at the end of life: a comparison of first generation black Caribbean and white populations." Palliative medicine 16(6): 540-541. | ***Quantitative non-randomized studies*** | yes | no | can't tell |
|  | 3.1. Are the participants representative of the target population? | Yes |  |  |
|  | 3.2. Are measurements appropriate regarding both the outcome and intervention (or exposure)? | Yes |  |  |
|  | 3.3. Are there complete outcome data? | Yes |  |  |
|  | 3.4. Are the confounders accounted for in the design and analysis? |  |  | can't tell |
|  | 3.5 During the study period, is the intervention administered (or exposure occurred) as intended? | N/A |  |  |
| Rawlings, D. and T. Glynn (2002). "The development of a palliative care-led memorial service in an acute hospital setting." International Journal of Palliative Nursing 8(1): 40-47. | ***Quantitative descriptive studies*** | yes | no | can't tell |
|  | 4.1. Is the sampling strategy relevant to address the research question? |  |  | can't tell |
|  | 4.2. Is the sample representative of the target population? |  |  | can't tell |
|  | 4.3. Are the measurements appropriate? | Yes |  |  |
|  | 4.4. Is the risk of nonresponse bias low? |  |  | can't tell |
|  | 4.5. Is the statistical analysis appropriate to answer the research question? | Yes |  |  |
| Robertson, M. J. P., A. Aldridge and A. E. Curley (2011). "Provision of bereavement care in neonatal units in the United Kingdom." Pediatric critical care medicine : a journal of the Society of Critical Care Medicine and the World Federation of Pediatric Intensive and Critical Care Societies 12(3): e111-115. | ***Quantitative descriptive studies*** | yes | no | can't tell |
|  | 4.1. Is the sampling strategy relevant to address the research question? | Yes |  |  |
|  | 4.2. Is the sample representative of the target population? | Yes |  |  |
|  | 4.3. Are the measurements appropriate? | Yes |  |  |
|  | 4.4. Is the risk of nonresponse bias low? | Yes |  |  |
|  | 4.5. Is the statistical analysis appropriate to answer the research question? | Yes |  |  |
| Rogers, C. and M. Greenfields (2017). "Hidden losses and ‘forgotten’ suffering: the bereavement experiences of British Romany Gypsies and Travellers." Bereavement Care 36(3): 94-102. | ***Qualitative studies*** | yes | no | can't tell |
|  | 1.1. Is the qualitative approach appropriate to answer the research question? | Yes |  |  |
|  | 1.2. Are the qualitative data collection methods adequate to address the research question? | Yes |  |  |
|  | 1.3. Are the findings adequately derived from the data? | Yes |  |  |
|  | 1.4. Is the interpretation of results sufficiently substantiated by data? | Yes |  |  |
|  | 1.5. Is there coherence between qualitative data sources, collection, analysis and interpretation? | Yes |  |  |
| Spruyt, O. (1999). "Community-based palliative care for Bangladeshi patients in east London. Accounts of bereaved carers." Palliative Medicine 13(2): 119-129. | ***Qualitative studies*** | yes | no | can't tell |
|  | 1.1. Is the qualitative approach appropriate to answer the research question? | Yes |  |  |
|  | 1.2. Are the qualitative data collection methods adequate to address the research question? | Yes |  |  |
|  | 1.3. Are the findings adequately derived from the data? | Yes |  |  |
|  | 1.4. Is the interpretation of results sufficiently substantiated by data? | Yes |  |  |
|  | 1.5. Is there coherence between qualitative data sources, collection, analysis and interpretation? | Yes |  |  |
