# Supplementary material for: Bereavement care for ethnic minority communities: A systematic review of access to, models of, outcomes from, and satisfaction with, service provision
Source: PLoS One. 2021 Jun 30;16(6):e0252188. doi: 10.1371/journal.pone.0252188 (PMC8244918; doi:10.1371/journal.pone.0252188)
Supplement: Attachment — Submitted filename: response to reviewers - 21st April 2021 CRM MA.docx [file pone.0252188.s004.docx]

| **Reviewer comments** | **Response** |
| --- | --- |
| **Editorial comments** | |
| 1. Please ensure that your manuscript meets PLOS ONE's style requirements, including those for file naming. The PLOS ONE style templates can be found at:  <https://journals.plos.org/plosone/s/file?id=wjVg/PLOSOne_formatting_sample_main_body.pdf> and  <https://journals.plos.org/plosone/s/file?id=ba62/PLOSOne_formatting_sample_title_authors_affiliations.pdf> | Thank you for the templates. We have formatted our paper using these. |
| 2. Please review your reference list to ensure that it is complete and correct. If you have cited papers that have been retracted, please include the rationale for doing so in the manuscript text, or remove these references and replace them with relevant current references. Any changes to the reference list should be mentioned in the rebuttal letter that accompanies your revised manuscript. If you need to cite a retracted article, indicate the article’s retracted status in the References list and also include a citation and full reference for the retraction notice. | We have reviewed our reference list to ensure it is complete and correct. Additional citations added when responding to reviewer comments can be identified in the tracked changes version of the manuscript. |
| 3. Thank you for stating the following in the Acknowledgments Section of your manuscript:  "There was no specific funding for this study. Dr Catriona Mayland and Matthew J Allsop are funded by Yorkshire Cancer Research."  We note that you have provided funding information that is not currently declared in your Funding Statement. However, funding information should not appear in the Acknowledgments section or other areas of your manuscript. We will only publish funding information present in the Funding Statement section of the online submission form.  Please remove any funding-related text from the manuscript and let us know how you would like to update your Funding Statement. Currently, your Funding Statement reads as follows:  "The author(s) received no specific funding for this work."  Please include your amended statements within your cover letter; we will change the online submission form on your behalf. | The funding information within the acknowledgement section of the manuscript has been removed.  We would like the Funding Statement to read,  ‘We did not receive any specific funding to undertake this research study. Dr Catriona R Mayland and Dr Matthew J Allsop are currently funded by the Yorkshire Cancer Research ‘CONNECTS’ Research Fellowship scheme (award references S406CM and L389MA respectively).  We have also provided details of the Yorkshire Cancer Research funding within the cover letter. |
| **4.** Please include captions for your Supporting Information files at the end of your manuscript, and update any in-text citations to match accordingly. Please see our Supporting Information guidelines for more information: <http://journals.plos.org/plosone/s/supporting-information>. | We have added captions at the end of the manuscript file. |
| **Reviewer 1** | |
| This article offers an important contribution in terms of identifying the absence of evidence to guide bereavement care and counselling for ethnic minority communities, and provides some justification of the need for further research and investment in participatory development of models of bereavement care. Minor revisions would strengthen and clarify this work, as outlined below. | Thank you for this feedback. |
| Introduction The justification for this review is well positioned at the outset in relation to the COVID-19 landscape. Some suggestions follow:  • Page 5, sentence beginning “Experiences of death and bereavement…” - perhaps insertion of “and are” (or similar) or a comma after “pandemic” would make this sentence clearer to read. | A comma has been added. |
| • Page 6: “only a small proportion are at risk of developing ‘complicated grief’…” from O’Connor et al (2014). Some explicit clarification of the meaning of 'complicated grief' as used in the context of this paragraph may be helpful here, ie briefly, how was risk specifically assessed to inform the model cited? More recent conceptualisations of the public health model in the context of bereavement have stratified levels of need according to measures of Prolonged Grief Disorder or PGD, rather than earlier framings and measures of 'complicated grief' eg Aoun et al, 2015 ‘Who Needs Bereavement Support? A Population Based Survey of Bereavement Risk and Support Need’. It may also be worth referring to this in some way, given the current ICD-11 also includes PGD rather than earlier conceptualisations of complicated grief. | We have changed the terminology from ‘complicated grief’ to ‘prolonged grief disorder.’ As the reviewer states, this is more in keeping with the ICD-11 classification and the illustrated public health model. Our reference list has been altered to reflect this.  ‘Only a small proportion are at risk of developing ‘prolonged grief disorder’ (PGD) which involves symptoms of separation anxiety and intense grief, as well as functional impairment, which endures for more than six months after the death.’ As part of this sentence we cite the following article:  Prigerson HG, Horowitz MJ, Jacobs SC, Parkes CM, Aslan M, et al. (2009) Prolonged Grief Disorder: Psychometric Validation of Criteria Proposed for *DSM-V* and *ICD-11*. PLOS Medicine 6(8): e1000121. <https://doi.org/10.1371/journal.pmed.1000121> |
| • It is valuable and helpful that the article includes acknowledgement of some of the limitations of the public health model. It would be pertinent to also recognise that recent iterations of the model define need according to risk of PGD and thus do not consider complex social/practical issues or other health/mental health concerns in this determination of need – which may be especially relevant given the disproportionate amplification of the risk of these issues among ethnic minority communities. | We have added the following text to further acknowledge this limitation:  ‘Additionally, the model has been developed based on the risk of developing PGD and doesn’t account for other important sociological or health factors which could impact on need for bereavement support.’  As part of this sentence, we have cited the following article:  Aoun SM, Breen LJ, Howting DA, Rumbold B, McNamara B, Hegney D (2015) Who Needs Bereavement Support? A Population Based Survey of Bereavement Risk and Support Need. PLoS ONE 10(3): e0121101. doi:10.1371/journal.pone.0121101 |
| • Page 7, definitions: I am assuming bereavement is being defined as post-death, however perhaps it would still be helpful to make this explicit given there is often some historical definitional confusion and conflation of terms (e.g. bereavement and grief) | This phrase, ‘after the death’, has been added for further clarity. |
| Results • Inclusion criteria states articles between 2000 and 2020 were included, however the Spruyt paper was published in 1999? | This was an error in the reporting of the timespan captured for searches. As shown in the search strategy in supplementary file 1, we did not limit the search from databases but only at the title/abstract review stage. We subsequently applied a limit by date to articles published between 1995 – 2020, to capture publications in the last 25 years. The manuscript has been updated to reflect this. |
| • Page 11: under quality appraisal, the insertion of an extra word may make the second sentence clearer – “…all applicable criteria were met for two studies (19,21), [?while] it was not possible to…” | The extra word has been added. |
| • In Table 1 and Table 2 the citation numbers appear to be incorrect. In manuscript and reference list, included studies were references 19-25. In tables, citations refer to references 18-24 which seems to be incorrect when looking at reference list. | This was due to an issue with the referencing software used during the preparation of the manuscript. This has now been amended. |
| • Page 19 – briefly, it may be helpful to refer to the nature of the “wider support” suggested for Romany Gypsy and Traveller communities. | We have clarified this as ‘wider, non-pharmacological support’. |
| • Page 20 – re: the barrier of culturally specific beliefs, perhaps another example (if possible, or was [22] the only relevant article here?) in addition to the example provided that pertained to Romany Gypsy and Traveller communities would thicken the description of this barrier. | This was the only article from our review which detailed culturally specific beliefs, although other articles alluded to these in a broader sense.  From our key stakeholder engagement, we are aware of other culturally specific beliefs although these weren’t found within our literature review. One example provided by our Patient and Public Involvement representative was the fact that Hindus within South Asian communities consider the bereaved to be ‘unclean’ for a ritual period. Hence, they are unable to participate in joyous festive occasions such as weddings during this time. This potentially limits the level of bereavement support available from family, friends and the wider community. |
| • Similarly on page 21, were there any other examples that might enrich the description of the two subthemes for ‘facilitators’? | The reporting of facilitators across the included studies was limited to those reflected in Figure 1. We have emphasised the lack of facilitators reported across the research literature in the discussion section of the manuscript. |
| • Figure 2 – it may be helpful to explicitly articulate the meaning of the arrows on either side of the pyramid on the conceptual map. | We have reviewed Figure 2 and revised the layout, removing the arrows. The arrows originally reflected the overarching themes relating to facilitators and barriers. We have revised the figure to embed the labels for the overarching themes in the boxes either side of the pyramid listing the identified barriers and facilitators. |
| Discussion The point that barriers at lower components may ultimately impact on awareness of and access to bereavement counselling is a valuable one. The call for participatory approaches to research related to ethnic minority communities is also important and well articulated.  • Page 23 – “…practical, legal and financial support may be needed and deemed more important by users during the bereavement period” – literature indicates these forms of support would probably be more significant in ‘early bereavement’ (vs say counselling, which is not often indicated or recommended until later time points). Perhaps it’s useful to clarify this rather than broadly state ‘bereavement period’ – which we know essentially isn’t characterised by a time frame. | Thank you for this feedback.  We have added in the term ‘initial bereavement period’ to provide further clarity. |
| • Page 24 – there are other examples of the nature of care experiences impacting upon bereavement and mitigating risk of complex outcomes which may be helpful to reference here to enhance/strengthen this point, eg Garrido, M.M. and Prigerson, H.G. (2014) The End of Life Experience – Modifiable Predictors of Caregivers’ Bereavement Adjustment, Cancer; Anderson, W. G., Kools, S., & Lyndon, A. (2013). Dancing around death: Hospitalist–Patient communication about serious illness, Qualitative Health Research; Ghesquiere, A. Marti Haidar, Y.M., Shear, K. (2011) Risks for Complicated Grief in Family Caregivers, Journal of Social Work in End of Life and Palliative Care (among others). | We have added two additional references to emphasise the relationship between care experiences before death and the subsequent impact on caregivers’ bereavement:  Wright, Alexi A, Zhang, Baohui, Ray, Alaka, Mack, Jennifer W, Trice, Elizabeth, Balboni, Tracy, Mitchell, Susan L, Jackson, Vicki A, Block, Susan D, Maciejewski, Paul K, and Prigerson, Holly G. "Associations Between End-of-Life Discussions, Patient Mental Health, Medical Care Near Death, and Caregiver Bereavement Adjustment." *JAMA : The Journal of the American Medical Association* 300.14 (2008): 1665-673.  Garrido, Melissa M, and Prigerson, Holly G. "The End‐of‐life Experience: Modifiable Predictors of Caregivers' Bereavement Adjustment." *Cancer* 120.6 (2014): 918-25 |
| • Page 24 – Should SPC services be urged to offer bereavement care spanning all three components? What about consideration of the way in which SPC services can partner/collaborate with/support other sectors, agencies and community groups engaged in service provision for ethnic minority communities, to invest in capacity building and support more holistic, comprehensive approaches to bereavement care across the spectrum for non-specialist needs (ie without being the lead provider of direct input across all components)? Would this be an implication that aligns with some of the public health literature and promotes quality of care and support? Further unpacking of some of the issues here may be helpful. | We agree that SPC services should not be the lead provider for all direct input and instead should adopt a collaborative approach to help both enable, and support, other services. There are examples of this type of model occurring e.g. using the ‘compassionate communities’ approach. We have revised this text to more clearly articulate this.  ‘Their role, however, should include forming partnerships with relevant agencies and community-based groups to help enable a more comprehensive network for bereavement support. This approach may help ‘bridge the gap’ in terms of practice and service delivery’.  We cite a recent paper outlining a compassionate communities approach to grief support: Zuniga-Villanueva G, Ramirez-GarciaLuna JL and Villafranca-Andino RI. A Compassionate Communities Approach in a Grief and Bereavement Support Program: Bridging the Gap in Palliative Care. *Journal of Social Work in End-of-Life & Palliative Care* 2021; 17: 9-18. DOI: 10.1080/15524256.2021.1894309. |
| • Page 26 – At the outset, the first and second sentence of second para might benefit from some tightening/clarification – eg who, is being argued, specifically needs to “align” with compassionate communities philosophy? | We have now specified at the start of this sentence that we are referring to ‘health and social care agencies’ and have expanded the sentence to provide further clarity.  ‘Health and social care agencies aligning with the philosophy and modelling of ‘compassionate communities’ would be another example of engagement to further enhance bereavement care.’ |
| • Page 26 – sentence starting “It is recognised, however that those not being able to speak…” – after “professionals”, insertion of extra words may assist readability/flow eg “[?and this] can lead to dissatisfaction…” | We have split this into two sentences to aid readability. |
| • Page 27 – the recent COVID-19 report from which the listed recommendations came needs to be referenced here. | Thank you, we have added this reference as suggested. |
| • Page 27 – if possible, brief further explanation may be helpful re: what is being suggested by an “intersectional approach,” and why this would be especially relevant here. | We have added an additional phrase to provide further clarity.  ‘…the identification of good practice in addressing the disparity of bereavement and loss support for BAME communities using an intersectional approach i.e. understanding further how social, economic and environmental aspects intersect with factors including gender, ethnicity and disability which can promote and disadvantage individuals.’ |
| **Reviewer 2** | |
| Reviewer #2: This review addresses bereavement care for ethnic minority communities in the UK, a woefully under-researched area of bereavement research. Overall, this a well conducted review and will provide a very useful contribution to the literature, highlighting the importance of the needs of ethnic minority communities in the context of the impact of COVID-19 pandemic. | Thank you for your feedback. |
| I suggest only a small number of revisions to aid with clarification, described below:  Introduction - It would be helpful to have in the Introduction a definition of ethnic minorities as the authors are using it in this review, in light of the increasing use of BAME in policy and media. Additionally some details on the context of what communities this might refer to in the UK would be helpful. | We have included a definition of ethnic minorities used throughout the review and cited the UK Government definition that was used.  Additionally, our key stakeholders gave specific input and feedback about the appropriate terminology to use within the manuscript. |
| - I wonder here whether reference to sociological/anthropological literature on death and bereavement in different cultures might be of use to put into context the gaps in research about the existing provision of bereavement support for ethnic minority groups e.g.  Death and Bereavement Across Cultures: Second edition edited by Colin Murray Parkes, Pittu Laungani, William Young Ahluwalia MK, Mohabir RK. Turning to Waheguru: Religious and Cultural Coping Mechanisms of Bereaved Sikhs. OMEGA - Journal of Death and Dying. 2019;78(3):302-313. doi:10.1177/0030222816688907 Dying, Death and Bereavement in a British Hindu Community By Shirley Firth Yasmin Gunaratnam (2008) From competence to vulnerability: Care, ethics, and elders from racialized minorities, Mortality, 13:1, 24-41, DOI: 10.1080/13576270701782969  Some of this literature may be a bit out of date now but that also reflects problem that understanding cultural practices in minority communities has been limited therefore it is unsurprising bereavement and mental health services continue to fail to meet the needs of communities. | This formed part of the discussion with our key stakeholders. Although we recognised there is sociological literature sharing learning about the huge cultural diversity towards attitudes and understanding about death and bereavement, we did not perceive we could include a wider discussion within the remit of this manuscript. We have highlighted the fact certain languages do not recognise the equivalent emotion of ‘grief’ from the English language. Additionally, we have added the following sentence into the discussion section (*Implications for research and practice*) to highlight the need for greater appreciation of different cultural practices.  ‘A wider appreciation of the different perceptions, meaning and context of death, dying and bereavement from diverse minority groups is required in order to build an evidence-base of research.’ |
| Methods - p.8-9 Some further details on inclusion and exclusion would be useful for example: was grey literature, commentaries/opinion pieces included? Why was the review limited to publications in past 20 years? | We have added further detail to the Data Sources and Searches section. We have made clear the inclusion of grey literature through the databases searched, and provided clarity on the inclusion of any study design providing primary data. We have also clarified why the literature was limited to the last 25 years (i.e. to reflect contemporary health service delivery structures across the UK). |
| - p.9 Was the full text review and data extraction also completed independently/in duplicate? | Data was extracted independently and this is now stated within the text. |
| - The patient and stakeholder engagement is a really important aspect of the study - could you say a little more about how patients and stakeholders were engaged in the conduct of the review and establishing the framework? | Each key stakeholder was asked to provide general feedback about the draft manuscript and the framework and also asked for advice about specific questions e.g. terminology to use about ethnicity. We think a succinct summary is provided about their input. We have added an additional sentence:  ‘Each key stakeholder also provided specific feedback to guide appropriate terminology. Engagement was conducted by a series of email dialogues and/or video meetings.’ |
| Results  The results are clear and well presented. One suggestion would be to explain the themes and subthemes further to explain what they mean and how they link together. For example the theme ‘unfamiliarity and irregularities’ is this referring to a ‘lack of awareness’ amongst healthcare/bereavement professionals of within bereaved communities or both? Likewise with ‘culturally specific beliefs’. | We have extended to the first sentence of this section to clarify this from the outset.  ‘The overarching theme was ‘unfamiliarity and irregularities’ which related to factors affecting healthcare professionals and/or bereaved ethnic minority communities.’  We haven’t added more detail to the ‘culturally specific beliefs’ as this is further expanded on within the results section. |
| Figure 2 is a really helpful diagram. | Thank you for this feedback. |
| Discussion - I wonder if the authors could offer any further explanation as to why these barriers exist/persist? Some suggestions are offered on p.24 and it is acknowledged that the reasons are complex. It may that the literature is simply too limited, but in terms of researchers and practitioners being able to take the findings forward, an analysis of some of the potential mechanisms, and not simply an identification of barriers and facilitators would be really valuable. | A key message arising from the review is the lack of literature and understanding to guide how best to move forward. This literature review is going to form the basis of the planned discussions with key stakeholder groups across the UK to explore how bereavement care can be improved for ethnic minority populations. Where possible we have provided some steer about how services might respond, such as the potential for specialist palliative care to extend reach through engagement and partnership with community-based groups (“Their role, however, should include forming partnerships with relevant agencies and community-based groups to help enable a more comprehensive network for bereavement support.”) |
| - It could also be helpful to put the findings in the context of bereavement care as a whole - to what extent are the challenges faced by ethnic minority groups unique and to what extent do they reflect a variable and under resourced sector? Clearly there are unique challenges and it would be helpful to spell these out a little further. | We are cautious, in view of the limited evidence, to expand our findings and discussion much further.  Previous reports about bereavement care *per se* have shown multiple barriers including insufficient tailoring of support and a lack of awareness of types of support available <https://www.healthwatch.co.uk/reports-library/pre-and-post-bereavement-support-exploring-barriers-universal-approach-end-life-care>  These issues could be applicable to many different individuals and groups.  Additionally, a previous report published in 2013 emphasised the requirement for policy makers and commissioners to understand the needs of ethnic minority populations to help ensure equitable health and social care.  <https://www.mariecurie.org.uk/globalassets/media/documents/who-we-are/diversity-and-inclusion-research/palliative-care-bame_full-report.pdf>  Rather than trying to further break down these challenges further, we think the manuscript’s current emphasis on co-design research and collaborative approaches to both understand and broach the challenges are the key messages we wish to convey. |
| - Finally, could the authors offer any explanation of why the evidence base is so limited? What's going on in UK bereavement research that this is the case? Broader citation of the sociological literature mentioned above might help answer this. | Generally, research funding for palliative and end-of-life care is sparse. Less than 0.3% of the £500 million spent on cancer research is allocated to palliative care (as outlined here: <https://spcare.bmj.com/content/6/1/2>)  Additionally, ethnic minorities are typically under-represented in health research, which is starting to be more acknowledged and recognised. Hence, we have added the additional line:  ‘In turn, research needs to focus, not just on what is needed, but robust evaluations of the efficacy and satisfaction with different models of care for both adults and bereaved children from ethnic minorities communities. Efforts are required to address the under-representation of those from an ethnic minority community in health research.’ |
